# Retraction: The structural and luminescence properties of plexcitonic structures based on Ag_2_S/l-Cys quantum dots and Au nanorods

**DOI:** 10.1039/d5ra90111a

**Published:** 2025-10-01

**Authors:** Irina G. Grevtseva, Oleg V. Ovchinnikov, Mikhail S. Smirnov, Aleksey S. Perepelitsa, Tamara A. Chevychelova, Violetta N. Derepko, Anna V. Osadchenko, Alexandr S. Selyukov

**Affiliations:** a Voronezh State University, Department of Optics and Spectroscopy Voronezh Russia Grevtseva_IG@inbox.ru; b Voronezh State University of Engineering Technologies Voronezh Russia; c Bauman Moscow State Technical University Moscow Russia; d P.N. Lebedev Physical Institute of the Russian Academy of Sciences Moscow Russia; e Moscow Institute of Physics and Technology Dolgoprudnyi Moscow Oblast Russia

## Abstract

Retraction of ‘The structural and luminescence properties of plexcitonic structures based on Ag_2_S/l-Cys quantum dots and Au nanorods’ by Irina G. Grevtseva *et al.*, *RSC Adv.*, 2022, **12**, 6525–6532, https://doi.org/10.1039/D1RA08806H.

The Royal Society of Chemistry hereby wholly retracts this *RSC Advances* article due to concerns with the reliability of the data.

There is a significant amount of overlap of text and images between this article and another published by the same authors.^1^ In addition, there is evidence of image manipulation in Fig. 2d and d′.

Given the significance of these concerns, the Editor has lost confidence that the findings presented in this paper are reliable.

The authors††The authors have informed us that Aleksey S. Perepelitsa is deceased and therefore has not been contacted regarding this retraction. were informed about the retraction of the article, and Irina Grevtseva informed us that all authors agreed to the retraction, but we have not heard from the remaining authors directly.

Signed: Irina Grevtseva on behalf of all authors

Date: 23rd September 2025

Retraction endorsed by Laura Fisher, Executive Editor, *RSC Advances*

**References**

1 I. Grevtseva, O. Ovchinnikov, M. Smirnov, A. Perepelitsa, T. Chevychelova, V. Derepko, A. Osadchenko and A. Selyukov, *Opt. Express*, 2022, **30**, 4668.

